# Ribosome heterogeneity in development and disease

**DOI:** 10.3389/fcell.2024.1414269

**Published:** 2024-07-17

**Authors:** Yuen Gao, Hongbing Wang

**Affiliations:** Department of Physiology, Michigan State University, East Lansing, MI, United States

**Keywords:** bioinformatics, cancer, development, macrophage, ribosome heterogeneity, stoichiometry

## Abstract

Traditionally viewed as a fixed and homogeneous machinery for protein synthesis, the ribosome is increasingly recognized for its heterogeneity, as indicated by emerging studies highlighting the functional relevance of specialized ribosomes. However, whether ribosome heterogeneity is merely an outcome limited to specific conditions or a pervasive cellular phenomenon remains unclear, and existing evidence on the extensive existence of ribosome heterogeneity is scant. Here, we leveraged existing proteomic data and employed ribosome ratio-omics (Ribosome^R^), which comprehensively analyzes ribosome protein stoichiometry across various biological samples exhibiting distinct functions, developmental stages, and pathological states. Using the 80S monosome proteomic data, Ribosome^R^ analysis unveils significant ribosome heterogeneity across different tissues, including fat, spleen, liver, kidney, heart, and skeletal muscles. Furthermore, examination of testes at various stages of spermatogenesis reveals distinct Ribosome^R^ signatures during tissue development. Analysis of the whole cell proteomic data finds that Ribosome^R^ undergoes dynamic changes during *in vitro* neuronal maturation, indicating functional associations with specific molecular aspects of neurodevelopment. In pathological contexts, Ribosome^R^ signatures in gastric tumors demonstrate functional links to pathways associated with tumorigenesis. Additionally, dynamic alterations in Ribosome^R^ are observed in macrophages following immune challenges. Collectively, our investigation across a diverse array of biological samples underscores the presence of ribosome heterogeneity, while previous studies observed functional aspects of ribosome specialization, in cellular function, development, and disease. The Ribosome^R^ barcode serves as a valuable tool for elucidating these complexities.

## Introduction

The ribosome, a macromolecular complex essential for protein synthesis, comprises approximately 80 highly conserved ribosome proteins (RPs) and four rRNAs in eukaryotes. Traditionally, ribosome composition was viewed as predominantly fixed and homogeneous, with dynamic regulation of protein synthesis primarily mediated by activity-dependent alterations in RP-mRNA interactions, post-transcriptional and post-translational modifications of mRNA and RP, and epigenetic effects from non-coding RNAs (Gebauer and Hentze, 2004; Song et al., 2021).

However, the discovery of ribosomes with distinct morphologies suggests potential heterogeneity in composition and function (Norris et al., 2021). Emerging evidence indicates that while some core RPs are consistently incorporated into ribosomes, ribosomes lacking specific RPs remain functional (Shi et al., 2017; Genuth and Barna, 2018). Furthermore, studies involving particular RPs have highlighted the functional relevance of specialized ribosomes. Mutations in specific RPs, such as *RPL5*, *RPL11*, *RPS17*, *RPS19*, and *RPS24*, have been associated with Diamond Blackfan Anemia (DBA) (Horos and von Lindern, 2012). It is hypothesized that ribosomes containing distinct RPs may preferentially translate specific pools of mRNAs. Notably, knockdown of *Rps19* or *Rpl11* leads to reduced translation of *Bag1* and *Csde1* mRNA, protein levels of which are diminished in DBA patient samples (Horos et al., 2012). Additionally, specialized ribosomes may translate mRNA pools linked to distinct cellular functions more effectively. For example, ribosomes containing RPS25 and RPL10A exhibit enrichment of non-overlapping mRNA pools associated with functionally diverse Gene Ontology (GO) groups (Shi et al., 2017).

Despite these insights, previous studies have primarily focused on the impact of individual RPs on ribosome function, leaving uncertainties regarding whether ribosome heterogeneity represents limited outcomes under specific conditions or a widespread cellular phenomenon. Moreover, evidence of extensive ribosome heterogeneity and its associations with cell identity, dynamic development, and pathogenesis remains limited. We hypothesize that functional ribosome heterogeneity involves alterations in numerous RPs on a genome-wide scale.

To explore this hypothesis, we employed a novel bioinformatics approach to examine the expression ratio of every RP. Our analysis, termed ribosome ratio-omics (Ribosome^R^), reveals specific heterogeneity associated with tissue type, *in vivo* and *in vitro* development, and disease. The Ribosome^R^ signature, serving as a genome-wide ribosome barcode, identifies a broad extent of ribosome heterogeneity and suggests its functional significance.

## Results

### Ribosome^R^ reveals tissue-specific ribosome heterogeneity

**FIGURE 1:**
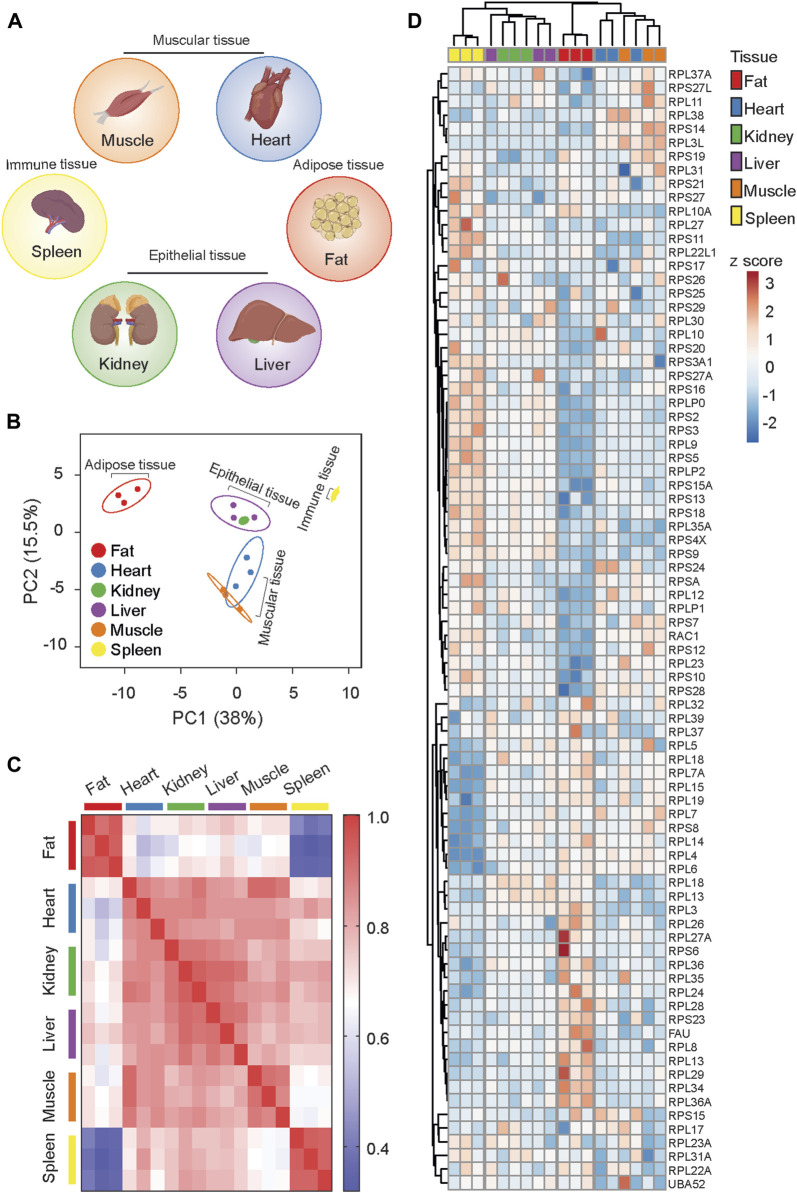
Ribosome^R^ reveals ribosome heterogeneity in tissues with distinct physiological functions. **(A)** Tissues with specialized function. **(B)** Principal component analysis of the expression ratio of each RP in six mouse tissues (n = 3 for each tissue). The Ribosome^R^ signatures of 82 RPs were compared. **(C)** Correlation matrix of the Ribosome^R^ signatures in six mouse tissues. Correlation coefficient of >0.85 is observed within each tissue. The correlation coefficient between different tissues is expressed as color-coded. **(D)** Heatmap analysis of the Ribosome^R^ signature in six mouse tissues. Rows (RP ratio) and columns (distinct tissues) are clustered using correlation distance and average linkage.

The functionality of tissues is theoretically determined by the expression of tissue-specific genes. However, as the level of mRNA transcripts often does not correlate positively with the corresponding protein levels, tissue-specific and activity-dependent alterations in translation provide necessary control over gene expression. Leveraging proteomic data on the 80S ribosome (Li et al., 2022), we analyzed the stoichiometry of core ribosome proteins (RPs) in various tissues. Specifically, we selected functionally distinct tissues: fat (adipose tissue), spleen (immune tissue), liver and kidney (epithelial tissues), and heart and skeletal muscle (muscle tissues) (Figure 1A). Our analysis revealed that each tissue possesses a distinct Ribosome^R^, providing a holistic signature of the stoichiometry of detected RPs (Supplementary Table S1). Principal component analysis (PCA) of Ribosome^R^ identified four functionally distinct groups: fat, kidney and liver, heart and skeletal muscle, and spleen (Figure 1B). Independent correlation analysis demonstrated that fat and spleen are distinctly separated from each other and from the other four tissues (Figure 1C). Additionally, the Ribosome^R^ heatmap unveiled four prominent clusters, with fat, spleen, kidney and liver, and heart and skeletal muscle each forming a distinct cluster (Figure 1D). Within the epithelial tissue cluster, kidney and liver showed partial separation, while within the muscle cluster, heart and skeletal muscle exhibited partial separation (Figure 1D). Our novel bioinformatic approach illustrates that genome-wide Ribosome^R^ detection unveils tissue-specific ribosome heterogeneity with functional relevance.

**FIGURE 2:**
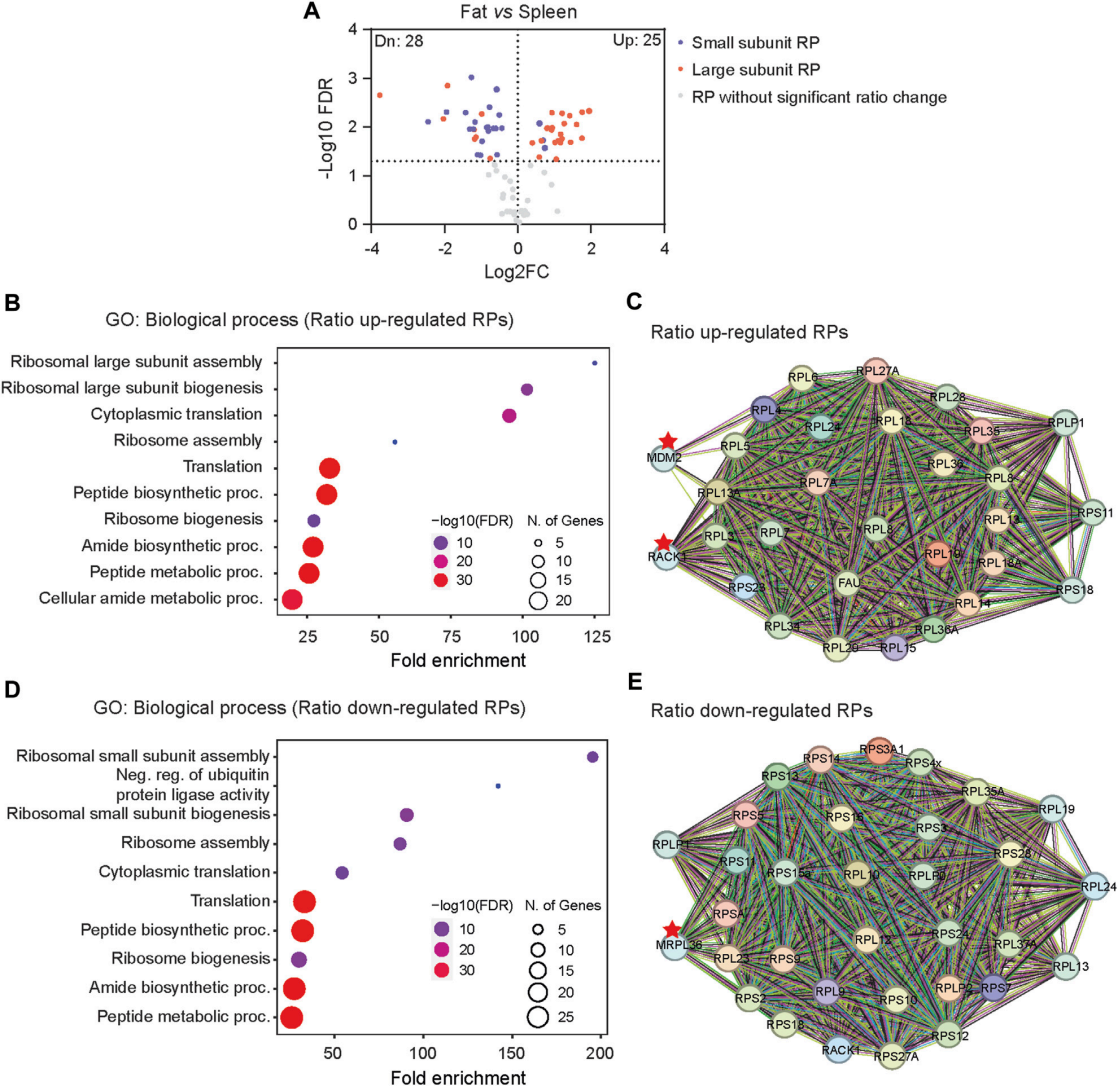
The distinct Ribosome^R^ in fat suggests potential functional relevance to adipogenesis. **(A)** Volcano plot identifies up- and downregulated expression ratios of specific RPs in fat *versus* spleen. **(B, D)** The top 10 biological processes identified by GO (gene ontology) analysis with the RPs showing upregulated **(B)** and downregulated ratios **(D)** in fat *versus* spleen. **(C, E)** STRING analysis identifies the molecular and functional interaction with the RPs that show increased **(C)** and decreased **(E)** stoichiometry in fat *versus* spleen.

We further compared Ribosome^R^ between fat and spleen, the most divergent tissues (Figure 1B), exhibiting the lowest correlation (Figure 1C). Interestingly, adipose tissue demonstrates a notably higher expression ratio predominantly in the large subunit RPs (Figures 2A–C) and a lower expression ratio in the small subunit RPs (Figures 2A, D, E). STRING analysis indicates functional interactions between MDM2 (murine double minute 2) and RACK1 (receptor for activated C kinase 1) and the RPs exhibiting an increased ratio in fat cells (Figure 2C). Furthermore, MRPL36, a mitochondrial RP, is predicted to interact with RPs showing a decreased ratio in fat (Figure 2E). Notably, RACK1 regulates adipogenesis (Kong et al., 2016), while MDM2 promotes the onset of fatty liver disease (Lin et al., 2022), both relevant to adipose tissue function.

We further explored whether the actual expression levels of RP, rather than the expression ratio, could detect ribosome heterogeneity. Utilizing the iBAQ values of each RP as described by Li et al. (2022), our analysis identified differences between fat and spleen tissues (Supplementary Figure S1A). Moreover, the analysis revealed that, except for one RP protein (RPL37), the expression levels of all other RP proteins were downregulated in fat tissue (Supplementary Figure S1B). The findings suggest that fat cells may exhibit an overall lower abundance of ribosomes compared to spleen cells (assuming that the same sample weight contains an equivalent number of cells). However, the stoichiometry of RPs is not revealed through analysis using the iBAQ values of each RP.

### Ribosome^R^ reveals dynamic ribosome heterogeneity in developing testis

**FIGURE 3:**
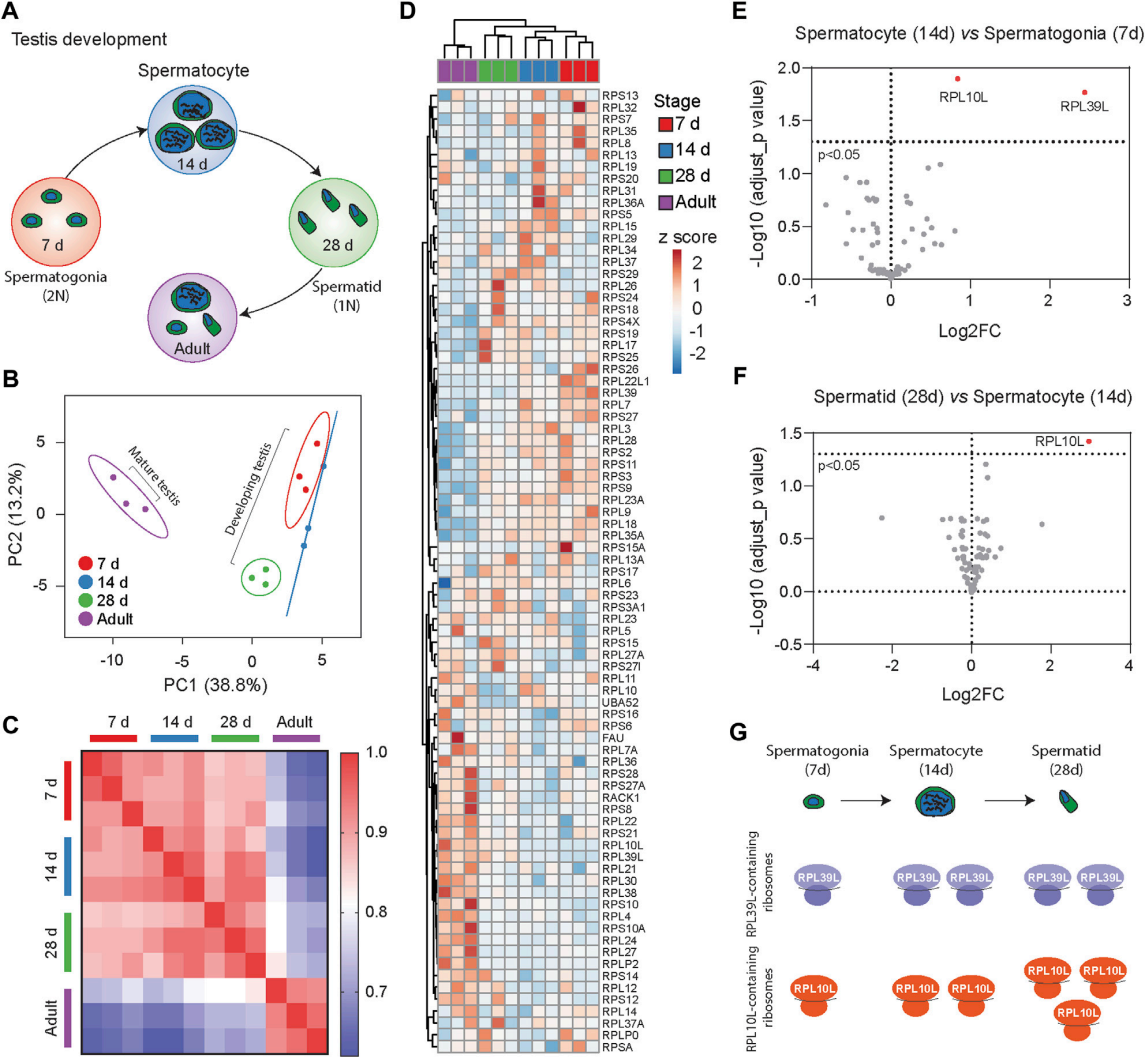
Ribosome^R^ reveals distinct ribosome heterogeneity during testis development. **(A)** Testes at different development stages display distinct cell types during spermatogenesis. **(B)** Principal component analysis of RP ratios in developing and mature mouse testes (n = 3 for each group). Ribosome^R^ signatures of 81 RPs were compared. **(C)** Correlation matrix of the Ribosome^R^ signatures in developing and mature mouse testes. Correlation coefficient of >0.91 is detected within each group. The correlation coefficient between different tissues is expressed as color-coded. **(D)** Heatmap analysis of the Ribosome^R^ signatures in developing and mature mouse testes. Rows are centered; unit variance scaling is applied to rows. Rows (RP ratio) and columns (7-day, 14-day, 28-day, and adult testis) are clustered using correlation distance and average linkage. **(E)** The volcano plot shows an upregulation in the expression ratios of RPL10L and PRL39L at day 14 compared to day 7 during testis development. **(F)** The volcano plot shows an upregulation in the expression ratio of RPL10L at day 28 compared to day 14 during testis development. (**G)** Analysis of Ribosome^R^ signatures suggests an increase in the number of RPL39L-containing ribosomes from spermatogonia to spermatocyte. The number of RPL10L-containing ribosomes rises from spermatogonia to spermatocyte and further increases from spermatocyte to spermatid.

To explore dynamic changes in ribosome heterogeneity during development, we analyzed the Ribosome^R^ of the 80S ribosome in mouse testes at various postnatal ages (Figure 3A). During spermatogenesis, the functional roles of testes vary with age. At 7 days postnatal, the testis supports spermatogonia genesis, characterized by 2N cells containing two copies of each chromosome. By 14 and 28 days, the testes support spermatocyte (4N cell) and spermatid (1N cell) development, respectively. The adult testis (∼60 days old) contains a mixture of these cells along with mature sperm cells. Ribosome^R^ analysis of proteomic data (Li et al., 2022) revealed development-specific heterogeneity (Supplementary Table S2). PCA of Ribosome^R^ segregated testes at different ages into four distinct groups (Figure 3B). Correlation analysis demonstrated stark differences in ribosome stoichiometry between adult testes and those in development at 7, 14, and 28 days of age (Figure 3C). The Ribosome^R^ heatmap depicted four distinct clusters (Figure 3D). Notably, hierarchical clustering revealed that the Ribosome^R^ signature in 7-day-old testes differed progressively from those at 14 days, 28 days, and adulthood (Figure 3D). We further compared the progressive alteration of Ribosome^R^ in spermatogonia, spermatocyte and spermatid (Figures 3E, F). The expression ratios of RPL10L and PRL39L are higher in spermatocyte than in spermatogonia (Figure 3E). The expression ratio of RPL10L is further increased in spermatid (Figure 3F). These findings underscore the association of progressive alterations in ribosome heterogeneity with distinct stages of testis development. Such progressive Ribosome^R^ alterations suggest an increase in the number of RPL39L-containing ribosomes from spermatogonia to spermatocyte. The number of RPL10L-containing ribosomes rises from spermatogonia to spermatocyte and further increases from spermatocyte to spermatid (Figure 3G). Interestingly, RPL10L is essential for meiotic processes (Jiang et al., 2017), while mutations in PPL10L lead to male infertility (Tu et al., 2020). Furthermore, deletion of RPL39L causes the mal-development of sperms with bent tails (Zou et al., 2021; Li et al., 2022), highlighting the indispensable role of RPL39L in spermiogenesis.

### Ribosome^R^ reveals dynamic ribosome heterogeneity during neuronal maturation

**FIGURE 4:**
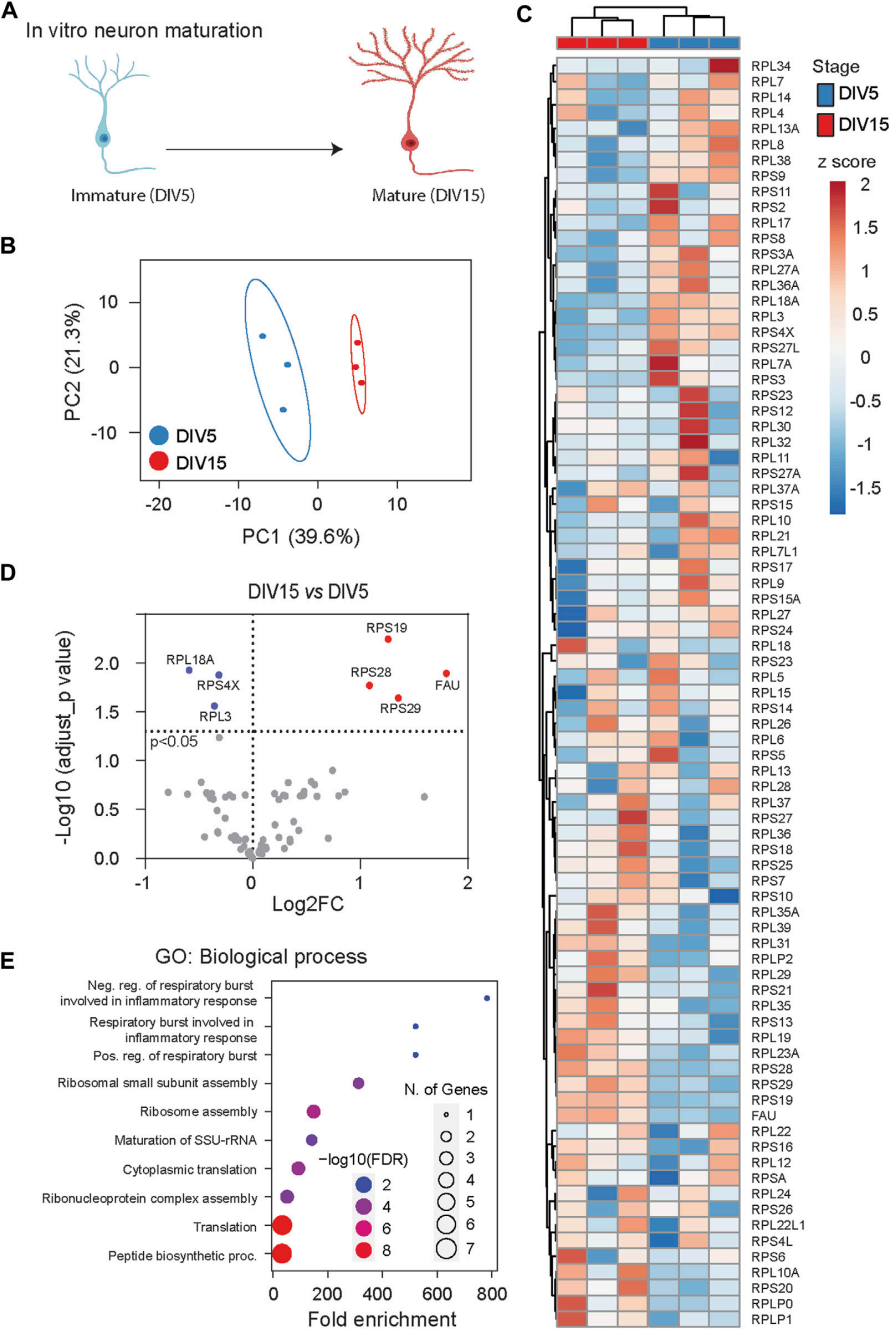
Ribosome^R^ reveals ribosome heterogeneity during *in vitro* neuron maturation. **(A)** Cultured cortical neurons undergo morphological and functional maturation. From DIV (days *in vitro*) 5 to DIV 15, axons and dendrites become elongated and branched; functional synapses are formed. **(B)** Principal component analysis of RP ratios in DIV 5 and DIV 15 neurons (n = 3 for each group). Ribosome^R^ signatures of 81 RPs were compared. **(C)** Heatmap analysis of the Ribosome^R^ signatures in DIV 5 and DIV 15 neurons. Rows (RP ratio) and columns (DIV 5 and DIV 15 neurons) are clustered using correlation distance and average linkage. **(D)** Volcano plot identifies upregulated and downregulated expression ratios of specific RPs during *in vitro* maturation. **(E)** The top 10 biological processes identified by GO (gene ontology) analysis with the RPs that show ratio alteration during *in vitro* maturation.

To elucidate dynamic changes in ribosome heterogeneity during the development of specific cell types, we analyzed Ribosome^R^ in cultured cortical neurons (Supplementary Table S3). In the well-characterized *in vitro* maturation process, neurons at day *in vitro* (DIV) five exhibit moderately developed axons and dendrites with limited functional synapses, while those at DIV15 demonstrate elaborated axons and dendrites, full-scale synaptogenesis, and robust expression of synaptic proteins (Dotti et al., 1988; Lesuisse and Martin, 2002; Zhou et al., 2009) (Figure 4A).

We examined the whole-cell lysate proteomic data collected from DIV 5 and DIV 15 neurons (Sharma et al., 2015). PCA of Ribosome^R^ revealed distinct ribosome compositions between immature DIV 5 neurons and mature DIV 15 neurons (Figure 4B). Heatmap analysis further highlighted distinct Ribosome^R^ signatures in DIV 5 and DIV 15 neurons as separate clusters (Figure 4C). Employing the differential Ribosome^R^ assay, we observed increased stoichiometry of four RPs and decreased stoichiometry of three RPs in mature neurons (Figure 4D). Gene ontology (GO) analysis with these seven RPs revealed that maturation-induced changes in ribosome heterogeneity are associated with respiratory burst among the top 10 biological processes (Figure 4E). Notably, respiratory burst is functionally relevant to the release of reactive oxygen species (ROS), dynamic alterations of which control synaptic plasticity and neural development (Massaad and Klann, 2011; Oswald M. C. et al., 2018; Oswald M. C. W. et al., 2018).

### Ribosome^R^ reveals ribosome heterogeneity in tumorigenesis

**FIGURE 5:**
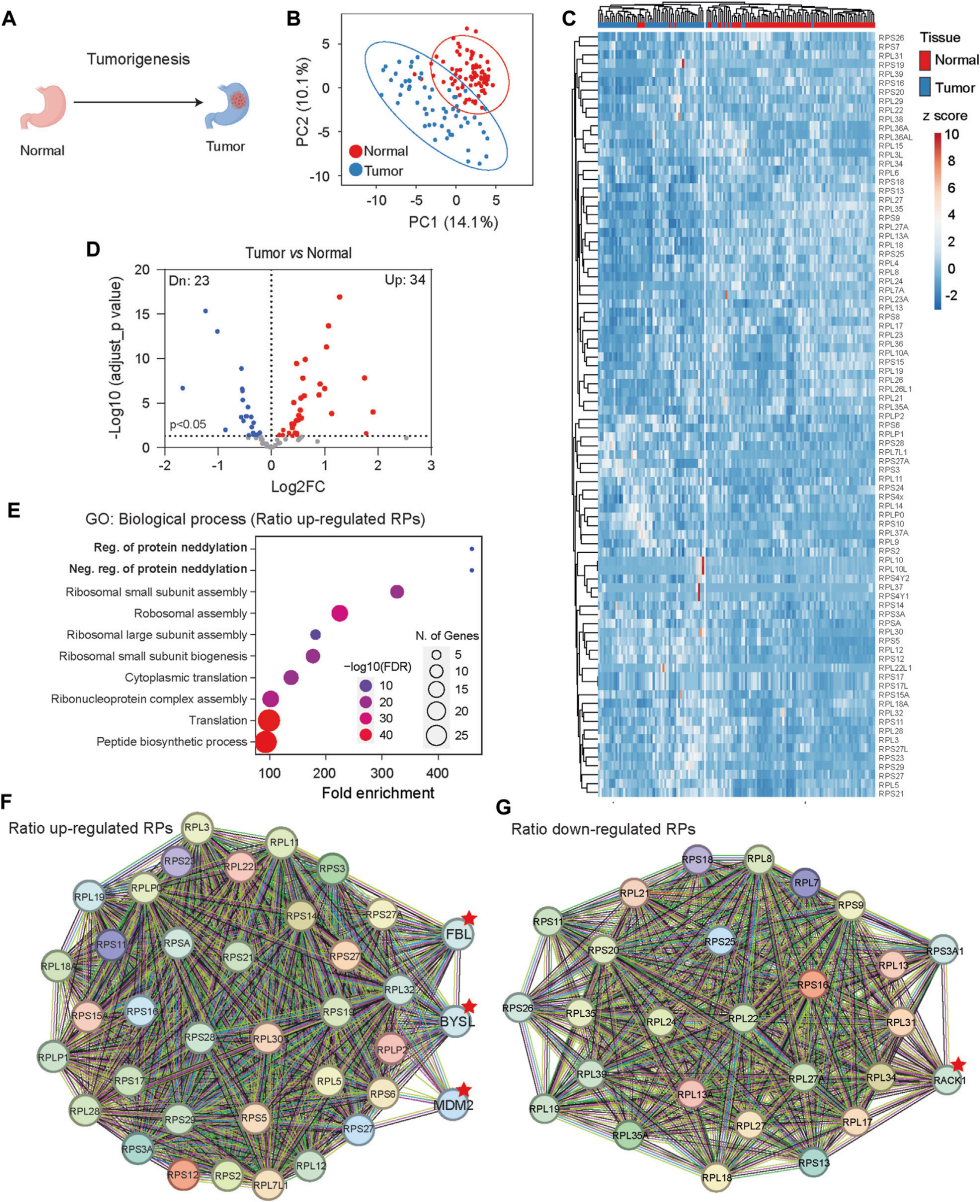
Gastric tissue tumorigenesis shows distinct Ribosome^R^ signatures. **(A)** Tumorigenesis alters cell fate and function in gastric tissue. **(B)** Principal component analysis of RP ratios in human normal gastric tissues (n = 82) and gastric tumor tissues (n = 58). Proteomic data from normal and tumor human gastric tissues were analyzed. The Ribosome^R^ signature of 86 RPs was compared. **(C)** Heatmap analysis of the Ribosome^R^ signatures in normal and tumor tissues. Rows (RP ratio) and columns (normal and tumor gastric tissues) are clustered using correlation distance and average linkage. **(D)** Volcano plot identifies up- and downregulated expression ratios of specific RPs in tumor tissue. **(E)** The top 10 biological processes identified by GO (gene ontology) analysis with the RPs that show ratio alteration in the tumor tissues. **(F, G)** STRING analysis and identification of the potential molecular and functional interaction with the RPs that show increased **(F)** and decreased **(G)** stoichiometry in the tumor tissues.

In comparison to normal tissue development, tumorigenesis is characterized by uncontrolled proliferation and cell immortalization. We investigated Ribosome^R^ signatures using the whole-cell lysate proteomic data collected from 82 human normal gastric tissues and 58 human gastric tumor tissues (Figure 5A) (Ni et al., 2019). PCA of Ribosome^R^ (Supplementary Table S4) revealed two partially separated groups (Figure 5B). Heatmap analysis unveiled two main clusters (Figure 5C). One cluster was enriched with normal gastric tissues (76 normal and 10 tumor tissues), while the other predominantly consisted of gastric tumor tissues (48 tumor and 6 normal tissues) (Figure 5C). In comparison with normal gastric tissues, tumor tissues exhibited increased expression ratios in 34 RPs and decreased ratios in 23 RPs (Figure 5D). GO analysis with these RPs identified neddylation among the top 10 biological processes (Figure 5E), potentially linking it to tumorigenesis (Zhou et al., 2019; Naik et al., 2020). Interestingly, among the two RPs associated with the Regulation of Protein Neddylation GO process, RPL11 could be neddylated and regulates the function of the tumor suppressor p53 (Sundqvist et al., 2009). RPL5 and RPL11 negatively regulate neddylation through interaction with MDM2 (Bailly et al., 2016). STRING prediction revealed that RPs with increased ratio changes interact with FBL (Fibrillarin), BYSL (Bystin or Bystin-Like), and MDM2 (Figure 5F), known to regulate tumorigenesis through p53 (Alarcon-Vargas and Ronai, 2002; Senturk and Manfredi, 2012). Additionally, STRING analysis with RPs showing decreased ratios suggested functional interactions with RACK1 (Figure 5G), known to regulate various aspects of ribosome function and tumorigenesis (Li and Xie, 2015).

### Ribosome^R^ reveals ribosome heterogeneity in acute macrophage activation

**FIGURE 6:**
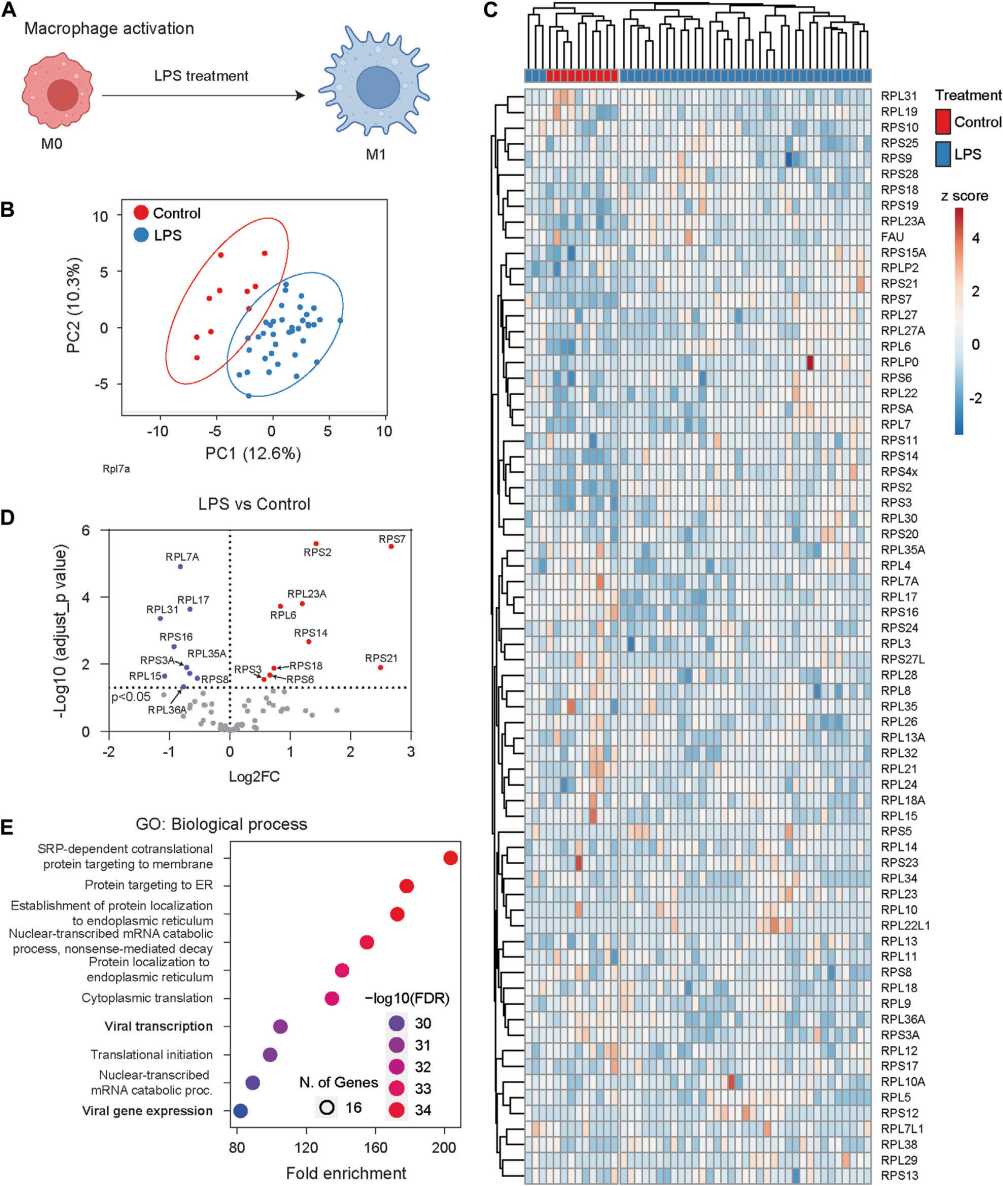
Macrophage cells undergo dynamic Ribosome^R^ changes following activation. **(A)** The proinflammatory activator LPS (lipopolysaccharide) stimulates macrophage cells, leading to immune responses such as the secretion of cytokines. **(B)** Principal component analysis of RP ratios in control (n = 10) and LPS-treated macrophages (n = 38). The Ribosome^R^ signature of 71 RPs was compared. **(C)** Heatmap analysis of Ribosome^R^ signatures in control and LPS-treated macrophage cells. Rows (RP ratio) and columns (control and LPS-treated cells) are clustered using correlation distance and average linkage. **(D)** Volcano plot identifies upregulated and downregulated expression ratio of specific RPs in the LPS-treated cells. **(E)** The top 10 biological processes identified by GO (gene ontology) analysis with the RPs that show ratio alteration in the LPS-treated cells.

Macrophages, as early-responding immune cells, play a pivotal role in reacting to infection or inflammation and neutralizing pathogens (Figure 6A) (Zhang and Wang, 2014). We analyzed single-cell proteomic data collected from the murine macrophage cell line RAW 264.7 (Woo et al., 2022). These RAW 264.7 cells were treated with lipopolysaccharide (LPS), a main component of bacterial membranes and a potent reagent for macrophage activation. We compared single-cell Ribosome^R^ signatures between 10 control cells and 38 LPS-challenged cells. The single-cell proteomic data provided reasonable coverage, detecting more than 50 RPs in each cell (Supplementary Table S5). PCA of Ribosome^R^ revealed two groups with limited overlap (Figure 6B). Heatmap analysis unveiled two main clusters (Figure 6C). Hierarchical clustering demonstrated that one main cluster contained all control cells in one sub-cluster and a small fraction of LPS-challenged cells (i.e., three cells) in another sub-cluster (Figure 6C). The other main cluster comprised the remaining LPS-challenged cells (i.e., 35 cells) (Figure 6C). Compared to the 10 control cells, the 38 LPS-challenged cells exhibited increased stoichiometry in 9 RPs and decreased stoichiometry in nine other RPs (Figure 6D). GO analysis revealed that among the top 10 biological processes, the dynamic changes in ribosome heterogeneity were functionally associated with responses to infection (Figure 6E).

## Discussion

Precise and dynamic regulation of gene expression is indispensable for cell function and development. The role of specific ribosome proteins (RPs) in mRNA translation and their association with pathology and disease underscore the proposed functional relevance of ribosome heterogeneity, although this remains a subject of debate. In this study, we conducted the first genome-wide and comprehensive characterization of ribosome composition in various tissues under physiological and pathological conditions. Using Ribosome^R^, we identified ribosome heterogeneity associated with tissue type and development, as well as alterations in Ribosome^R^ under disease conditions. Our findings underscore the broad existence of ribosome heterogeneity and its functional significance. Moreover, we anticipate that Ribosome^R^, as an effective analytical method, will be instrumental in unraveling an essential and novel dimension of gene expression regulation.

Previous studies have noted a suggestive differential expression of specific ribosome protein (RP) mRNAs across various mammalian tissues (Bortoluzzi et al., 2001; Gupta and Warner, 2014; Guimaraes and Zavolan, 2016). Approximately 25% of RP genes exhibit tissue-specific mRNA expression (Guimaraes and Zavolan, 2016), although this observation is also debated (Gupta and Warner, 2014). The functional relevance of mRNA heterogeneity remains unclear, as mRNA expression profiles do not consistently segregate into distinct PCA groups based on tissue function. Hierarchical clustering analysis reveals that functionally similar tissues are not necessarily clustered together but rather exhibit some degree of separation. For instance, the RP mRNA signature in kidney (an epithelial tissue) shows greater similarity to adipose tissue than to liver (another epithelial tissue) (Guimaraes and Zavolan, 2016). In a study by Sugihara et al., ribosome proteomic data from liver, testis, and mammary gland revealed differential levels of several RP-like proteins but failed to detect tissue-specific ribosome heterogeneity (Sugihara et al., 2010). In our study, we utilize Ribosome^R^ and, for the first time, identify functionally relevant and tissue-specific heterogeneity. Adipose, epithelial, muscular, and immune tissues exhibit distinct Ribosome^R^ patterns. However, we observe that Ribosome^R^ may not adequately detect significant heterogeneity among tissues of similar types. For instance, heart and skeletal muscle, as well as liver and kidney, exhibit slightly overlapping Ribosome^R^ patterns. It remains unclear whether the presence of mixed cell types within each tissue compromises the resolution of Ribosome^R^. Comparing Ribosome^R^ between two tissues with markedly different physiological functions—fat and spleen—we identify striking differences in ribosome composition. Our Ribosome^R^ analysis further suggests the functional relevance of tissue-specific heterogeneity, exemplified by the involvement of RACK1 and MDM2 in adipogenesis and fatty liver disease.

If ribosome heterogeneity is indeed associated with biological function, it is reasonable to expect that ribosome composition in a specific tissue dynamically adjusts to functional changes during development. Even within tissues comprising different cell types, Ribosome^R^ reveals the interaction of development and function across distinct developmental stages. For instance, in testis tissue, Ribosome^R^ captures differences across four distinct development stages. Similarly, in cultured primary cells primarily composed of neurons rather than mixed cell types, Ribosome^R^ distinguishes between immature and mature neurons. The ribosome changes associated with maturation detect gene ontology (GO) functions related to reactive oxygen species (ROS) production. Notably, ROS not only influences neural development and maturation (Wilson et al., 2018) but also exhibits excessive production in Diamond-Blackfan anemia (DBA) (Rio et al., 2019). Intriguingly, among the four ribosome proteins showing an increased ratio in mature DIV 15 neurons, RPS19, RPS28, and RPS29 mutations have been identified in DBA (Horos and von Lindern, 2012; Dokal et al., 2022). Causally, deficient RPS19, mutations of which are most frequent in DBA (Draptchinskaia et al., 1999), directly leads to anemia in mice (Debnath et al., 2017).

Ribosome alterations have been implicated in cancer and tumorigenesis (Guimaraes and Zavolan, 2016; Kang et al., 2021). Targeting ribosomes represents a potential avenue for cancer therapy (Gilles et al., 2020). Guimaraes and Zavolan analyzed The Cancer Genome Atlas (TCGA) data and found that certain ribosome protein (RP) genes, such as *RPL39L*, are commonly dysregulated in different cancer types; however, whether cancer cells and normal cells exhibit distinct ribosome heterogeneity remains unclear (Guimaraes and Zavolan, 2016). Our Ribosome^R^ analysis, conducted with a large proteomic dataset comprising 82 normal and 58 gastric tumor tissues, revealed two separate clusters enriched with normal and tumor samples, respectively. Among the 34 RPs exhibiting an increased ratio in cancer, missense mutations in *RPL11* and *RPL5* occur in 73% and 66% of the 19,000 cancer samples across 49 cancer types (Orsolic et al., 2020). While an increase in RPS15A associates with the progression of various cancers (Guo et al., 2018), a decrease in RPS15A inhibits cancer cell proliferation (Zhao et al., 2015; Yao et al., 2016). Among the 23 RPs showing a decreased ratio in cancer, heterozygous deletion of *RPL22* is observed in approximately 10% of T-acute lymphoblastic leukemia (T-ALL) cases (Rao et al., 2012). Various mutations in *RPL22* are also found in solid cancers (Ferreira et al., 2014).

While it remains unclear whether tumor-specific ribosome heterogeneity regulates the translation of tumor-specific proteins or reflects a pathological outcome, STRING prediction suggests functional associations of RPs with an increased ratio with the MDM2/p53 axis, FBL, and BYSL. Among the Ribosome^R^ -identified tumor-associated RPs with an increased ratio, RPL5, RPL11, RPS3, RPS14, RPS19, RPS27, RPS27A, and RPS27L have been found to physically interact with MDM2 (Kang et al., 2021), which inhibits the function of the tumor suppressor p53 (Alarcon-Vargas and Ronai, 2002; Senturk and Manfredi, 2012). Beyond its established role in regulating transcription, the MDM2/p53 axis, which affects and responds to nucleolar activity (Liu et al., 2016), may also participate in the ribosome assembly process. The interaction between RPs and FBL suggested by Ribosome^R^ may imply altered ribosome biogenesis in gastric cancer (Nguyen Van Long et al., 2022). BYSL, involved in rRNA processing and 40S ribosome biogenesis during development and cancer cell proliferation (Adachi et al., 2007; Wang et al., 2009), could play a role in cancer progression.

STRING prediction suggests that RACK1, which functionally interacts with RPs showing a decreased ratio, stably associates with ribosomes (Johnson et al., 2019) and regulates the translation of specific mRNA pools (Majzoub et al., 2014; Thompson et al., 2016). Intriguingly, RACK1 affects cancer progression in a tissue-dependent manner; while it promotes breast and lung cancers (Li and Xie, 2015), it suppresses gastric tumors (Deng et al., 2012).

Single-cell Ribosome^R^ analysis unveils dynamic ribosome alterations in macrophages following immune challenge. Intriguingly, Ribosome^R^ responses to lipopolysaccharide (LPS) are not uniform across all cells. The three LPS-challenged cells exhibit distinct Ribosome^R^ signatures compared to both the control cells and the remaining 35 LPS-challenged cells. This observation aligns with the emerging understanding that macrophages undergo epigenetic diversification (Ma et al., 2022). The different single-cell Ribosome^R^ signatures in LPS-stimulated macrophages further suggest functional diversity.

In summary, Ribosome^R^ identifies the broad existence of ribosome heterogeneity with functional relevance to development and disease. This analytical approach provides a novel strategy to assess ribosome composition and function.

## Limitation of the study

The Ribosome^R^-identified heterogeneity in different tissues and during *in vivo* testis development relies on proteomic data obtained from purified 80S ribosomes. It's important to acknowledge that active translation primarily occurs via polyribosomes, albeit a recent study discovered that the 80S monosomes handle local mRNA translation at neuronal synapses (Biever et al., 2020). At present, it remains unclear whether polyribosomes and the 80S monosomes possess distinct ribosomal protein compositions.

The Ribosome^R^-identified heterogeneity during *in vitro* neuronal maturation, gastric tumorigenesis, and macrophage activation relies on proteomic data from whole-cell lysates of bulk tissues or single cells. It is uncertain if all the ribosomal proteins (RPs) detected within the whole-cell extracts are assembled into functional ribosomes. Thus, it is reasonable to suggest that the current Ribosome^R^ analysis offers a remodeling potential of ribosome composition. Future experiments aimed at gathering proteomic data from purified polyribosomes and analyzing ribosomal protein expression ratios are crucial for advancing our understanding of the significance of ribosome heterogeneity.

We anticipate that Ribosome^R^ will introduce a robust new analysis approach for detecting ribosome heterogeneity using current data. Moreover, we hope it will stimulate future validation efforts when polyribosome data becomes available.

## Experimental procedures

### Datasets

We used the published public proteomic data. The relative expression ratio of RPs in various tissues was analyzed with proteomic data of isolated 80S monosomes from fat, spleen, liver, kidney, heart, and skeletal muscle (Li et al., 2022). The relative expression ratio of RPs in developing testes was analyzed with proteomic data of isolated 80S monosomes from 7-day, 14-day, 28-day, and adult testes (Li et al., 2022). The relative expression ratio of RPs in cultured cortical neurons (Sharma et al., 2015) and human normal and tumor gastric tissues (Ni et al., 2019) was analyzed with whole-cell proteomic data. The relative expression ratio of RPs in control and activated macrophage cells was analyzed with single-cell proteomic data (Woo et al., 2022). The whole-cell extracts were obtained after sonication in lysis buffers containing detergent (2% SDS), reducing agent (5, or 10 or 100 mM DTT) at high temperatures (99°C, or 95°C, or 70°C) followed by alkylation with 10 mM iodoacetamide (Sharma et al., 2015; Ni et al., 2019; Woo et al., 2022).

### Ribosome ratio-omics (Ribosome^R^)

We calculated the expression ratio of each RP to the level of all detected RPs. Depending on the isolation and proteomic detection method, most of the core RPs were detected. Data from various tissues and developing testes reported the expression of 82 and 81 RPs, respectively (Li et al., 2022). Data from cultured cortical neurons (Sharma et al., 2015), human normal and tumor gastric tissues (Ni et al., 2019), and control and activated macrophage cells (Woo et al., 2022) reported the expression of 81, 79, 86, and 71 RPs, respectively. The iBAQ algorithm was used for the ribosomal protein ratio analysis because the iBAQ values are proportional to the molar quantities of the proteins (Woo et al., 2022). The expression ratio of each RP was calculated by (individual RP iBAQ value)/(total RPs iBAQ value). The holistic ribosome ratio-omic (Ribosome^R^) values are reported in Supplementary Tables S1–S5.

### Principal component analysis (PCA)

The principal component analysis was performed using ClustVis based on the pcaMethods R package (Metsalu and Vilo, 2015). Unit variance scaling is applied to rows; SVD (singular value decomposition) with imputation is used to calculate principal components. Prediction ellipses are such that, with a probability of 0.95, a new observation from the same group will fall inside the ellipse.

### Correlation analysis

The correlation matrix, which captures the interrelationships among different samples, was computed utilizing the Pearson correlation coefficient within the R statistical environment. Subsequently, the correlation results were visually presented through plots generated using GraphPad Prism 9 software.

### Heatmap clustering analysis

The Heatmap clustering analysis was performed using ClustVis (Metsalu and Vilo, 2015) based on the pheatmap R package (version 0.7.7). Rows are centered; unit variance scaling is applied to rows. Both rows and columns are clustered using correlation distance and average linkage.

### Differential Ribosome^R^ analysis and volcano plot

The two-tailed t-tests were performed to identify the RPs showing significantly different expression ratios. The Benjamini-Hochberg correction method was applied to adjust the obtained *p* values. A discernible difference in RP expression ratios is deemed significant if the adjusted *p* value falls below the conventional threshold of 0.05. The identified RPs are presented in the volcano plots utilizing GraphPad Prism 9 software, enabling visualization of the magnitude and significance of the observed changes in RP ratios.

### Gene ontology (GO) analysis

The GO analysis and enrichment were performed using ShinyGO (Ge et al., 2020) with a false discovery rate (FDR) cutoff set at 0.05. The top 10 biological process terms were extracted and presented. The relevance of biological processes is listed in descending order based on their FDR significance and fold enrichment.

### STRING analysis

The STRING database was used to predict potential molecular interactions with the RPs, which showed ratio alterations. Analysis was conducted using the default parameters within the STRING database. The RPs with altered expression ratios are placed in the center shell. The interacting proteins are placed in the outer shell.

### Illustration graphs

Illustration graphs were made with BioRender.

## One-sentence summary

Ratio-omics signature of ribosome deciphers functionally relevant heterogeneity in development and disease.

## Data Availability

The raw MS data of human stomach tissues presented in the study are deposited in the proteomeXchange via the iProX partner repository, accession number PXD011821.

## Supplementary material

SUPPLEMENTARY TABLE S1 Expression ratio of RPs in different mouse tissues of fat, heart, skeletal muscle, kidney, liver, and spleen.

SUPPLEMENTARY TABLE S2 Expression ratio of RPs in mouse testes at different ages.

SUPPLEMENTARY TABLE S3 Expression ratio of RPs in DIV5 and DIV15 neurons.

SUPPLEMENTARY TABLE S4 Expression ratio of RPs in human normal and tumor gastric tissues.

SUPPLEMENTARY TABLE S5 Single-cell expression ratio of RPs in control and LPS-treated RAW 264.7 cells.
