# Supplementary material for: Ribosome heterogeneity in development and disease
Source: Front Cell Dev Biol. 2024 Jul 17;12:1414269. doi: 10.3389/fcell.2024.1414269 (PMC11288964; doi:10.3389/fcell.2024.1414269)
Supplement: Supplementary file 3 [file DataSheet1.PDF]

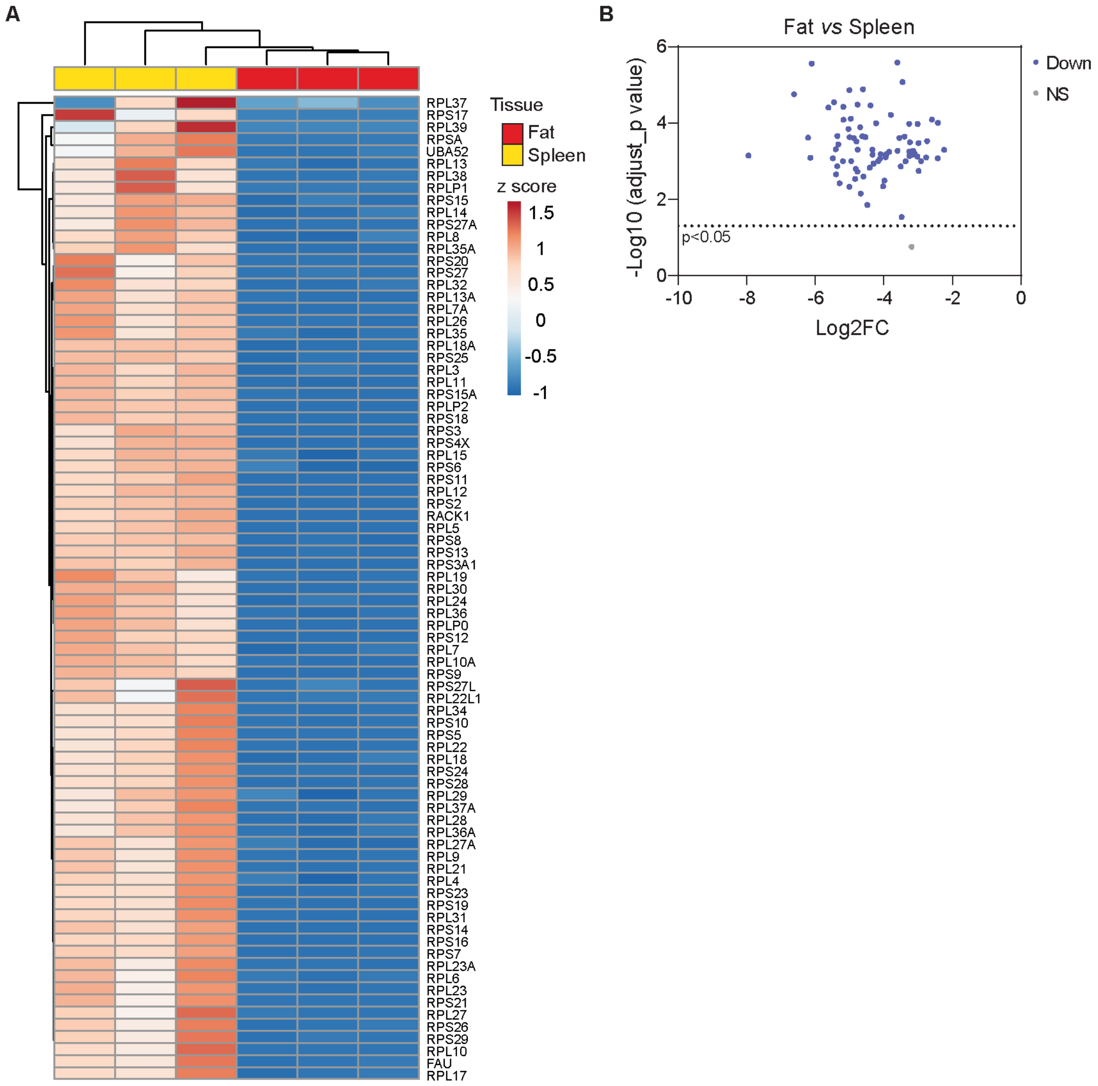

Supplementary Figure 1. Comparison of ribosome protein level between fat and spleen. The iBAQ values of each RP detected in fat and spleen are compared. (**A**) Heatmap analysis was conducted to assess the levels of ribosomal proteins in mouse fat and spleen tissues using iBAQ values. Rows are centered; unit variance scaling is applied to rows. Rows and columns are clustered using correlation distance and average linkage. (**B**) The volcano plot shows that there are only down-regulated but not up-regulated expression levels of specific RPs in fat *vs* spleen tissue.
